# Pneumonia in Transplant Recipients: A Comprehensive Review of Diagnosis and Management

**DOI:** 10.7759/cureus.73669

**Published:** 2024-11-14

**Authors:** Ramakanth Pata, Joanna Kristeva, Bhanu Kosuru

**Affiliations:** 1 Pulmonary and Critical Care Medicine, One Brooklyn Health, New York, USA; 2 Pulmonary and Critical Care Medicine, University of Cincinnati Medical Center, Cincinatti, USA; 3 Internal Medicine, One Brooklyn Health, New York, USA; 4 Internal Medicine, University of Pittsburgh Medical Center (UPMC) East, Monroeville, USA

**Keywords:** bacterial pneumonia, fungal pneumonia, hematopoietic stem cell transplantation, immunosuppressive therapy, invasive pulmonary aspergillosis, pneumonia, solid organ transplantation, viral pneumonia

## Abstract

Transplant recipients have an increased risk of complications, including graft dysfunction and infections, which can be life-threatening if not recognized early. Pneumonia ranks as one of the most frequent complications in both solid organ and hematopoietic stem cell transplants. Clinical symptoms manifest late during infections in immunocompromised patients. An aggressive approach centered on early confirmatory diagnosis and a low threshold for empiric therapy is often the most effective strategy. The isolation of a pathogen in an upper airway sample does not necessarily mean the same organism is responsible for pneumonia. Viruses such as CMV (cytomegalovirus virus) may function as co-pathogens for opportunistic infections in transplant recipients in addition to causing their own primary infectious syndrome. Furthermore, some viruses exhibit immunomodulatory effects that can affect the graft function. Given the exhaustive list of causative pathogens responsible for pneumonia, the best approach to the diagnosis is to have a conceptual framework that includes a detailed history, such as the type of transplant, degree of immunosuppression, antimicrobial prophylaxis, risk factors, time of presentation since transplantation and the radiographic pattern on the CT chest (computer tomography of the chest). Management depends predominantly on the degree of antimicrobial resistance, drug-to-drug interaction, and adjustments to the immunosuppression.

## Introduction and background

Organ transplantation has garnered significant global attention and practice, substantially improving morbidity and mortality rates for millions afflicted by end-stage organ failure or hematologic disorders. Significant advancements in surgical techniques, effective immunosuppressive therapies, and comprehensive preventive measures during the transplant process have collaboratively enhanced patient outcomes and reinforced the evidence supporting the efficacy of transplantation [1].

Solid organ transplants offer patients the opportunity to live without extracorporeal treatments, such as dialysis for renal failure or Extracorporeal membrane oxygenation for end-stage heart failure, thereby substantially enhancing their quality of life. Furthermore, hematopoietic stem cell transplants provide an aggressive therapeutic option, offering potential cures for conditions such as leukemia and lymphoma. Nevertheless, it is imperative to acknowledge that transplant recipients may encounter various complications that can pose serious risks to their health and survival; many of these complications can be life-threatening if not urgently addressed. Despite advancements in immunosuppressive therapies aimed at reducing the risk of organ rejection, significant challenges in achieving favorable transplant outcomes persist, particularly due to infectious complications and inadequate graft survival. In fact, infections remain the leading cause of mortality among lung transplant recipients, highlighting the critical necessity for diligent monitoring and management strategies in transplant care [2].

Generally, organ transplantation is classified into two categories: hematopoietic stem cell transplants and solid organ transplants. This classification is essential due to the substantial variability in associated risk factors, potential complications, and management strategies between these categories. For instance, the nature of infectious complications differs based on the type of transplant, emphasizing the need for specialized care pathways tailored to each patient group [3].

Overview of hematopoietic stem cell transplant

Hematopoietic stem cell transplant (HSCT), commonly referred to as a bone marrow transplant, is a complex procedure requiring healthy stem cells to successfully engraft in the recipient’s bone marrow. These stem cells can originate from various sources, including the donor’s bone marrow, peripheral blood, or umbilical cord blood. Among these sources, peripheral blood stem cells are particularly noted for facilitating faster neutrophil engraftment. However, stem cell engraftment is associated with a heightened risk of graft-versus-host disease (GVHD), in contrast to stem cells derived from umbilical cord blood. While umbilical cord blood cells typically result in a slower reconstitution of B and T cell immune responses, this may adversely affect overall recovery [4].

To promote effective cellular engraftment, the recipient's existing bone marrow must be rendered non-functional. This is accomplished through a conditioning regimen, which typically involves cytotoxic chemotherapy and, in certain cases, total body radiation. These high-intensity conditioning protocols referred to as “myeloablative regimen” aim to mitigate the likelihood of cancer relapse; however, they may also increase organ toxicity. This type of conditioning prolongs the period of neutropenia and therefore heightens the risk of complications, such as mucosal barrier disruption, thus increasing vulnerability to infections [5].

Furthermore, the incorporation of anti-thymocyte globulin into the conditioning regimen significantly reduces T cell counts, thereby increasing the risk of severe infections, particularly invasive fungal infections, and herpes simplex virus (HSV) [6]. For older patients, a “non-myeloablative” regimen is typically recommended, which features a lower intensity of treatment to mitigate potential organ toxicity during recovery.

HSCT is classified as either autologous or allogeneic based on the source of the infused stem cells. Autologous HSCT involves the collection of stem cells from the patient's own blood, whereas allogeneic HSCT utilizes cells from a related or unrelated donor. The engraftment process post-infusion generally takes approximately two to three weeks. The patient remains vulnerable to infection during this period until the full reconstitution of immune system. Therefore the risk of infection gradually decreases over time since the transplantation. It is imperative to note that B and T cell reconstitution occurs more rapidly in autologous HSCT compared to allogeneic HSCT. This disparity can result in a higher incidence of invasive fungal and herpes infections in patients undergoing allogeneic HSCT [7]. Additionally, the use of unrelated or mismatched donors may introduce further challenges, such as delayed immune reconstitution, complicating recovery. Interestingly, in certain scenarios of allogeneic HSCT, long-term immunosuppression may not be even necessary, particularly in the absence of indications of graft-versus-host disease [8]. This customized approach is vital for developing treatment plans aimed at optimizing patient outcomes while minimizing potential complications.

Overview of solid organ transplant

In solid organ transplants, immunosuppression is particularly intense immediately following the procedure, and patients are placed on varying lifelong immunosuppression regimens to prevent rejection. These regimens depend on numerous factors, including the type of organ transplanted and the timing involved. For instance, transplants of the small intestine, heart, and lungs often necessitate more aggressive immunosuppression, particularly in HLA (human leukocyte antigen) mismatched cases, compared to kidney and liver transplants [9]. Another consideration is the organ harvested from the deceased versus living donors. For example, Kidney transplant recipients from deceased donors receive induction T cell depletion therapy, unlike those from living donors [9].

Several factors can elevate the risk of infection post-transplant. The duration since the transplant and the degree of immunosuppression primarily influence the likelihood of both infectious and non-infectious complications. Infection risk also depends on the underlying disease necessitating the transplant. For instance, patients undergoing prolonged steroid treatment for multiple myeloma or therapy with purine analogs for chronic lymphocytic leukemia face an increased likelihood of infection [10]. HLA mismatched or haplo-identical (partially mismatched HLA) allografts further elevate the potential for infectious complications [11].

A conceptual framework for assessing infection risk in transplant recipients is termed the “Net state of immunosuppression.” This framework encompasses several factors, including the timing and dosage of immunosuppression regimens, underlying diseases, and co-morbidities such as diabetes, uremia, and concomitant viral infections like CMV (cytomegalovirus), EBV (Epstein-Barr virus), and HHV-6 (human herpesvirus-6). Various tests have been proposed to evaluate immunocompetence levels, but few have been validated to accurately predict infection risk. Blood levels of torque teno virus (TTV) are emerging as a potential biomarker for infection risk, particularly in kidney allograft recipients, as TTV levels inversely correlate with immunocompetence [12]. Some cell sorting technologies even measure specific pathogen-driven T and B cell immune responses to assess immunity [13].

Overview of CAR-T cell therapy

Chimeric antigen receptor T cell therapy (CAR-T cell therapy) has gained prominence for treating hematological malignancies, achieving remission rates of 50-80%. It involves the infusion of genetically altered T cells containing a fusion protein that integrates an antigen-binding domain with T cell signaling proteins, along with co-stimulatory molecules [14]. These cytotoxic T cells can be either autologous (derived from the patient) or allogeneic (from a donor) obtained via leukapheresis. Infusions typically occur after lympho-depletion employing fludarabine and cyclophosphamide. Despite its success rate, CAR-T cell therapy can lead to significant complications such as cytokine release syndrome, hemophagocytic lymphohistiocytosis, and infectious complications, including bacterial or viral infections. Notably, fungal infections post-CAR-T therapy are relatively rare [15]. Persistent pancytopenia, particularly absolute neutropenia, raises infection risk for up to one year following treatment. Although some guidelines recommend against routine antibacterial prophylaxis, certain centers may prescribe fluoroquinolones in cases of severe neutropenia. Antiviral, anti-PJP (*Pneumocystis jerovecii* pneumonia), and anti-fungal prophylaxis are generally advised [16].

Pulmonary complications following transplant

In transplant populations, pulmonary complications can develop swiftly, necessitating a heightened level of vigilance since immunosuppressive therapies may diminish inflammatory responses and obscure clinical signs. Consequently, maintaining a low threshold for aggressive intervention upon the slightest clinical or radiological indicators can prevent critical clinical decline. It is noteworthy that hospitalized patients with solid organ transplants, aside from lung and heart transplants, may experience lower mortality rates than non-transplant patients with sepsis and bacteremia. This phenomenon is likely attributable to the reduced inflammatory response or the implementation of more immediate and aggressive treatment [17].

Pneumonia is one of the common infectious complications seen in post-transplant care. Given the immunosuppressive state, the suspicion, diagnosis, and treatment of pneumonia must be approached with consideration of the duration since the transplant, the level of immunosuppression, and the severity of the case [18].

When managing post-transplant pneumonia, it is essential to emphasize certain principles, as outlined below.

Principles

Clinical symptoms manifest late during infections in immunocompromised patients. Therefore, an aggressive approach centered on early confirmatory diagnosis and a low threshold for empiric therapy is often the most effective strategy [19].

Viruses may function as co-pathogens for opportunistic infections and can impact various processes in the post-transplantation phase. Some of these viruses, which cause indirect effects alongside their primary infectious syndrome, include CMV, HBV (hepatitis B virus), HCV (hepatitis C virus), EBV, RSV (respiratory syncytial virus), HHV-6, and adenovirus. For instance, CMV infection leads to pneumonitis, adrenalitis, and vasculitis, as well as neutropenia, creating susceptibility to numerous opportunistic infections. CMV infections alter inflammatory cascades and suppress scavenger pathways, increasing vulnerability to *Aspergillus *and *Pneumocystis* infections [20]. Moreover, CMV reactivation heightens the risk of graft rejection, requiring a more robust immunosuppression regimen, which can increase the risk of opportunistic infections [21]. Occasionally, CMV infections may make patients more susceptible to HHV-6 infections and vice versa. CMV is not the sole immuno-modulatory virus; RSV infections can also predispose patients to *Aspergillus* infections. In some cases, secondary EBV infections elevate the risk of post-transplant lymphoproliferative disorders, typically B cell lymphomas [22]. Community-acquired respiratory pathogens increase the likelihood of bacterial infections and graft rejection, especially in lung transplant recipients [22]. Thus, it is crucial to test for multiple pathogens.

Depending on the severity and urgency of infections, adjustments to immunosuppression may sometimes be necessary. However, this must be approached with caution as it may lead to graft rejection or immune reconstitution syndrome [23].

It is important to note that prophylaxis in transplant patients does not completely eliminate the risk of infection; it merely postpones its onset [24].

Involvement of infectious disease specialists may lead to lower mortality and readmission rates, but interestingly it could also lengthen the hospital stay [25].

Many transplant recipients may harbor organisms that exhibit antimicrobial resistance. Selecting antimicrobial therapy should consider the potential for drug resistance, drug interactions, and organ toxicity [26,27].

Neutropenia typically heightens the risk of bacterial and fungal infections, while T cell immune deficiencies predispose individuals to viral and intracellular infections (e.g., CMV, mycobacterial infections) [28]. Additionally, steroids elevate the risk of PJP and *Aspergillus* infections [29]. Belatacept, a CTLA-4 inhibitor used in kidney transplantation, raises the likelihood of EBV-related lymphoproliferative disorders, PJP, and CMV infections [30]. Furthermore, therapeutic aphaeresis for kidney transplantation (in the setting of ABO incompatibility) can increase susceptibility to encapsulated organisms like *Pneumococcus*, *Haemophilus influenzae*, and *Neisseria meningitidis* [31,32].

The next sections will address the diagnosis and treatment of infectious pneumonia in recipients of both solid organ transplants and hematopoietic stem cell transplants. This overview will highlight the most prevalent pathogens and, whenever applicable, a distinction in approach will be made between HSCT and solid organ transplant recipients.

## Review

Timing of pulmonary complications

Hematopoietic Stem Cell Transplantation

The occurrence of both infectious and non-infectious pulmonary complications varies depending on the time since HSCT. Accordingly, this timeline is divided into three distinct phases. While the duration of each phase can differ for each individual, this framework serves as a useful guideline for differential diagnosis. Phase 1 corresponds to the time until neutrophil recovery, whereas the subsequent phases relate to the reconstitution of B and T cell immunity [33]. Generally, the risk of infection diminishes after six months post-HSCT, particularly when the absolute CD4 count exceeds 200 cells/µL or the lymphocyte count is above 500 cells/µL. Infection risk is present throughout all three phases in allogeneic recipients, while it is primarily observed during the initial two phases following autologous HSCT [34].

Phase 1 (pre-engraftment period; 0-30 days): The pre-engraftment period refers to the time before transplanted stem cells successfully engraft, until neutrophil recovery. This process usually takes two to three weeks for healthy stem cells to integrate into the recipient's bone marrow. Consequently, this stage is marked by significant neutropenia [35]. Neutropenic fever is the primary clinical symptom during this time, with an infectious source identified in only about 20-30% of cases [36]. Most fevers are caused by aerobic gram-positive and gram-negative bacteria, prompting the use of antibiotic prophylaxis, particularly for high-risk patients, especially those who have undergone myeloablative regimens. Persistent fever during this phase might also be due to *Candida *or *Aspergillus* infections, more often in the latter half of this period [35].

Fungal and bacterial pneumonia typically present as nodular patterns or localized infiltrates on radiographic images. In contrast, conditions like viral infections or other non-infectious issues, such as hemorrhagic alveolitis or cytokine-related engraftment syndrome, usually appear as diffuse infiltrates [37].

Engraftment syndrome is identified by an erythematous maculopapular rash, fever, weight gain, and diffuse infiltrates, particularly characterized by bilateral ground glass opacities, peri-bronchial consolidation, and interlobular septal thickening. Diagnosis can be confirmed through a skin biopsy of the rash or by identifying diffuse alveolar damage on a lung biopsy. It’s important to note that engraftment syndrome occurs much more frequently in autologous HSCT than in allogeneic HSCT [38].

Hyper-acute graft-versus-host disease (GVHD) shares clinical features with engraftment syndrome, presenting with rash, abdominal cramps, and diarrhea, especially in cases of HLA mismatch [39].

Diffuse alveolar hemorrhage is generally diagnosed by observing progressively increasing returns in sequential bronchoalveolar lavage and finding over 20% hemosiderin-laden macrophages. During the pre-engraftment phase, diffuse infiltrates mainly arise from non-infectious causes [40].

Phase 2 (early post-engraftment period; 30-100 days): The early post-engraftment period, spanning from 30 to 100 days, is characterized as a high-risk interval for the development of acute GVHD; consequently, many patients receive immunosuppressive therapy for its management. This phase is marked by impaired cellular immunity, as complete reconstitution of cellular or humoral immune functions may extend over several months to years following engraftment [41].

Pneumonia emerges as a prevalent complication during this period. The radiographic presentation, including nodular versus diffuse infiltrates, aids in refining the differential diagnosis. Predominantly, bacterial and mold infections manifest as localized infiltrates or nodular patterns, with invasive aspergillosis accounting for approximately 40-50% of nodular pneumonia cases during this phase [42]. Other mold infections observed in this period include mucormycosis, particularly when presenting with sinus symptoms, or *Fusarium* in the context of skin lesions. There have been rare reports of infections due to *Mycobacterium* and *Nocardia* [37]. Diffuse infiltrates are often associated with PJP, viral infections such as CMV or respiratory viruses, or non-infectious complications like Idiopathic pneumonia syndrome, a form of pulmonary toxicity that can occur following myeloablative regimens in allogeneic HSCT [33,43].

Other non-infectious pulmonary complications during this period may encompass delayed pulmonary toxicity syndrome, pulmonary venous-occlusive disease, and cryptogenic organizing pneumonia [44].

*Pneumocystis* pneumonia should be suspected in patients exhibiting dry cough, fever, and an interstitial pattern on chest radiographs featuring diffuse infiltrates, particularly those on suboptimal prophylaxis or non-adherent to their prophylactic regimen [45].

Respiratory viruses, including SARS-CoV-2 (severe acute respiratory syndrome coronavirus 2), may lead to both upper and lower respiratory tract infections, presenting as either diffuse or localized infiltrates. In cases of pneumonia, the detection of respiratory viruses in upper respiratory samples does not necessarily indicate that the same pathogen is responsible for lung involvement, as co-infections with pathogens such as *Aspergillus*, *Pneumocystis*, or CMV are well-documented. Therefore, comprehensive diagnostic evaluations, including bronchoscopy and biopsy, should be diligently pursued [46].

Cytomegalovirus should be contemplated in high-risk patients presenting with diffuse infiltrates during this phase who are not undergoing anti-CMV prophylaxis [47].

In patients with cutaneous exposure to contaminated soil, disseminated *Strongyloides* must always be considered, as it can result in a potentially fatal hyper-infection syndrome manifesting as acute respiratory distress syndrome [48]. Disseminated toxoplasmosis is also observed during this phase, characterized by diffuse infiltrates in high-risk populations such as those living in tropical countries or who eat undercooked meat or shellfish [49].

Phase 3 (late post-engraftment period; > 100 days): The late post-engraftment period, usually starting three months after HSCT, is marked by reduced cellular and humoral immunity, increasing the risk of opportunistic infections, especially following allogeneic HSCT. Additionally, treatment for chronic GVHD can further suppress the immune system, due to delayed immune reconstitution. Common infections during this phase include CMV, VZV (varicella-zoster virus), EBV (leading to post-transplant lymphoproliferative disorder), and *Nocardia*. The likelihood of CMV infection is highest in seropositive recipients who have seronegative donors [47]. Respiratory infections from encapsulated bacteria such as *Pneumococcus*, *Haemophilus influenzae*, and *Neisseria meningitidis* can also occur in this stage [33].

Nocardia primarily causes nodular infiltrates but may also appear as skin lesions or cerebral infarcts [50]. Invasive mold infections and PJP occur in patients on steroids for chronic GVHD.

Non-infectious pulmonary complications can include chronic GVHD, presenting as bronchiolitis obliterans syndrome, and pulmonary veno-occlusive disease from pulmonary cytolytic thrombi. The post-transplant lymphoproliferative disorder is related to EBV and typically presents during this period as nodular infiltrates. Instances of mycobacterial infections, including pulmonary and extra-pulmonary tuberculosis, have been documented in this phase, often presenting various pulmonary infiltrates [41].

It’s important to note that this timeline serves only as a conceptual guide for differential diagnosis. Patients remain susceptible to community-acquired pathogens, such as seasonal viruses or endemic fungal infections, depending on their exposure risk throughout their lives. Thus, these factors should always be considered during clinical assessments.

In summary, HSV infection mainly occurs during the pre-engraftment phase, while CMV and VZV infections are more common post-engraftment. Among fungal infections, *Candida* is more frequently seen pre-engraftment, whereas PJP is primarily observed during the post-engraftment phase.

Solid Organ Transplantation

Recipients of solid organ transplants undergo significant immunosuppression right after transplantation, often including steroids and T cell-directed therapies. Lifelong immunosuppression is typically required to prevent organ rejection [9]. The timeline following solid organ transplantation is generally divided into three phases [51,52].

Nosocomial infections are most prevalent during the first month post-transplant. For the next six months, the risk of opportunistic or reactivation infections is at its highest. After six months, community-acquired infections tend to dominate the late post-transplant period. Care should be taken, as intensifying the immunosuppression regimen to address graft rejection effectively resets the timeline to the initial phase [53].

Phase 1 (immediate post-transplant period; < 1 month): During this period, surgical complications, primary graft dysfunction, and nosocomial infections are common. Donor-related bacterial and fungal infections may also occur. Many nosocomial or donor-derived infections could exhibit antibiotic resistance [52]. Utilizing bronchoscopy-alveolar lavage with gram stain might facilitate early infection identification, directing appropriate antibiotic treatment. A gram-positive coccus suggests the potential presence of vancomycin-resistant *Enterococci* or methicillin-resistant *Staphylococcus aureus *(MRSA), while a gram-negative bacillus may indicate multi-drug-resistant Pseudomonas or Legionella [54].

*Candida* or HSV infections can also occur during this phase. In the nosocomial, it is crucial to expect azole resistance or non-albicans *Candida *if pseudo-hyphae appear in the BAL (bronchoalveolar lavage) specimen. Patients with cystic fibrosis face risks of developing bacterial or *Aspergillus* infections if they were previously colonized before the transplant, as typical pre-surgical prophylaxis may not cover some pathogens [2].

Phase 2 (early post-transplant period; 1-6 months): Intensive immunosuppression is at its peak during this phase, leading to opportunistic infections of *Listeria*, *Mycobacterium*, *Aspergillus*, *Pneumocystis*, CMV, EBV, VZV, *Toxoplasma*, and *Strongyloides *[51,52]. Geographic exposure can increase susceptibility to endemic fungal infections of *Histoplasma* (Ohio and Mississippi River valleys), *Coccidioides* (Southwest US), *Paracoccidioides* (Central and South America), *Cryptococcus*, and *Blastomyces* (Mid West, South-central, and Southeastern United States). High-risk patients may experience *Nocardia* infections for up to four months and cryptococcal infections for up to six months post-solid organ transplant [20].

Phase 3 (late post-transplant period; > 6 months): Most patients stabilize with lowered immunosuppression levels in this phase. Community-acquired infections such as seasonal respiratory viruses, *Pneumococcus*, endemic fungi, and Legionella are more prevalent, alongside noninfectious complications [52]. These infections are generally more severe than those in non-transplant patients. There is an increasing prevalence of non-tuberculous mycobacterial infections during this time [55]. Patients who received earlier prophylaxis may experience late CMV infections in this phase. Those facing graft rejection may have increased risks of CMV or PJP infections due to heightened immunosuppression, especially if prophylaxis has been halted [56].

CAR-T Cell Therapy

The recovery period after CAR-T cell therapy can be categorized into four distinct phases: (i) day 0 refers to the date of CAR infusion; (ii) from induction up to day 0 is the immediate post-induction phase; (iii) from day 0 to day 30, non-infectious complications are most prevalent; (iv) from day 30 to six months, infectious complications become the primary concern, and (v) sixs months to one year, infectious complications continue to be observed [57].

During the initial 30 days, non-infectious complications such as cytokine release syndrome, immune effector cell-associated neurotoxicity, and hemophagocytic lymphohistiocytosis occur. Infections typically emerge after the first month, with the heightened risk lasting for up to a year, largely influenced by pancytopenia and absolute neutropenia. Inpatient mortality linked to CAR-T cell therapy is primarily due to infections, rather than from non-infectious complications. The clinical signs of cytokine release syndrome and sepsis can be strikingly similar, and unfortunately, CRP or procalcitonin are not reliable indicators for antibiotic treatment in these patients [57, 58].

Epidemiology, risk factors, and clinical presentation

Key factors to consider when obtaining a medical history include the patient's medical background, transplant details, and exposure history. Beyond co-morbidities, medical history should encompass recent hospitalizations and any current or past microbial colonization. The transplant history ought to detail the type of transplant received, the time elapsed since the procedure, the level and type of immunosuppression, any rejection episodes, and current antimicrobial prophylaxis. For instance, a T cell depletion protocol not only heightens the risk for herpes viruses and invasive fungal infections but also increases susceptibility to neutropenia-associated infections [59].

The exposure history should address seasonal risk factors, local epidemiology, and personal exposure risk due to travel or contact with sick individuals. In certain geographic regions, endemic fungal infections might be suspected. A number of infections can result from the reactivation of organisms acquired before the transplant, particularly in cases of systemic mycosis, strongyloidiasis, tuberculosis, herpes viruses, etc. [60]. Therefore, past travel or exposure history can be significant for assessing exposure risks. A history of CMV infection can elevate the risk of invasive aspergillosis or PJP pneumonia, possibly by causing changes in cellular immunity [56].

Common symptoms like fever, cough, shortness of breath, hemoptysis, and pleuritic chest pain, prevalent among the general public, may be absent in transplant recipients. Consequently, clinicians should be particularly vigilant with diagnostic testing. Fever is generally non-specific, with non-infectious causes representing 22% of cases. Conversely, the absence of fever does not rule out infection, as up to 40% of infections in post-transplant patients may not present with fever. The clinical presentation can be vague, with manifestations limited to altered mental status, unexplained hypotension, isolated fever, or even subtle findings on imaging. Infection must be a top consideration in differential diagnoses when a post-transplant patient presents with these isolated symptoms. Furthermore, pursuing an aggressive diagnostic workup is necessary in nearly all cases as opposed to adopting a conservative stance [61].

Community-Acquired Respiratory Viruses

Community-acquired respiratory viruses encompass influenza A/B, RSV, parainfluenza virus, adenovirus, metapneumovirus, rhinovirus, enterovirus, and human coronaviruses [62]. These viruses typically start as upper respiratory infections, manifesting symptoms like fever, cough, sore throat, and rhinorrhea; they may progress to acute respiratory failure, severe hypoxia, and shortness of breath in certain cases. Not all patients exhibit standard symptoms; for instance, a multi-center study found that only two-thirds of transplant recipients with influenza experienced fever, and less than half reported myalgias [63]. Consequently, typical symptoms often seen in immunocompetent hosts may not appear, prompting clinicians to maintain a high degree of clinical suspicion. Unlike other viruses, adenoviruses and herpesviruses generally result from the reactivation of latent infections rather than new infections. While rhinovirus is often benign in immunocompetent individuals, it can cause severe pneumonia in allogeneic HSCT and lung transplant recipients [64,65]. SARS-CoV-2 infection may additionally have leukopenia and lymphopenia [66].

Herpes Viruses

These include CMV, HSV, VZV, EBV, and HHV-6 [67].

Cytomegalovirus (CMV) is the most prevalent viral infection in the post-transplant period. It can cause direct multi-system infections and may act as a co-pathogen or risk factor for bacterial and fungal superinfections. In lung transplant patients, CMV infection is often the risk factor for bronchiolitis obliterans syndrome. CMV can emerge as either reactivation or disease, primarily presenting as pneumonia. Although reactivation of the latent virus in transplant recipients is the most common cause of CMV disease, instances of passive transmission via lung allograft have been reported. Primary infection occurs when seronegative recipients acquire CMV from a seropositive donor [47].

The incidence of CMV pneumonia, particularly in the early post-transplant phase, has dropped following the introduction of preemptive prophylaxis like Ganciclovir or Foscarnet. Historically, prior to prophylactic regimens, reactivation rates were approximately 70% in seropositive allogeneic recipients and 40% in autologous HSCT. In this group, a third of allogeneic recipients developed CMV disease, while around 5% of autologous HSCT recipients did. Currently, the incidence of CMV pneumonia is reported to be between 1-5%. Late CMV reactivation in the post-engraftment period for allogeneic HSCT remains a concern, with infection rates of 15-30% and disease rates of 6-18%. Clinicians must consider CMV if the initial diagnostic work-up yields negative results [33,68].

The highest risk for CMV pneumonia in HSCT recipients is found in CMV seropositive individuals with seronegative donors or cord blood transplants. In solid organ transplants, the greatest risk appears in seronegative recipients with seropositive donors, particularly for lung and small bowel transplants due to high-intensity immunosuppression [69]. Prophylaxis with ganciclovir, valganciclovir, or letermovir (a novel agent targeting DNA terminase complex) has reduced the risk of CMV infection, although does not obviate the likelihood of late reactivation. Mismatched or partially matched transplants further heighten the risk of CMV reactivation due to delayed restoration of CMV-specific T cell responses. Other risk factors for CMV infection include GVHD, high-dose steroid therapy, and previous CMV viremia.

Typically, CMV infection occurs within one to three months following transplantation, though it can be delayed in those receiving antiviral prophylaxis. The clinical presentation of CMV infection ranges from subclinical to life-threatening organ-invasive disease, typically categorized into three groups: (i) Subclinical infection: marked by viremia/antigenemia or virus shedding from the respiratory or urinary tract; asymptomatic virus shedding from the respiratory tract can happen without tissue invasion; (ii) Mononucleosis-like syndrome: presents with fever, malaise, and leukopenia; (iii) Organ invasive disease: affecting various organs such as the gastrointestinal tract, retina, lungs, or CNS (CMV pneumonia presents with fever, shortness of breath, cough, and hypoxemia).

Considering the high mortality and morbidity associated with CMV infections in the post-transplant phase, prevention is often the most effective strategy to mitigate complications. Ideally, seronegative donors and seronegative blood transfusions should be prioritized for seronegative organ recipients, especially in solid organ transplants. Given that a significant portion of the donor pool is seropositive, this approach might lead to extended waiting times. An alternative prevention strategy involves pharmacological prophylaxis with ganciclovir, which can delay the onset and reduce the incidence and severity of the infection. For this reason, two prophylaxis methods are commonly implemented: “universal prophylaxis”, which involves at least three months of ganciclovir treatment at the time of transplantation if either the donor or recipient is seropositive, and “preemptive prophylaxis”, where ganciclovir is administered if asymptomatic viremia is detected through serial p65 testing or PCR (polymerase chain reaction) testing [70].

Epstein-Barr virus (EBV) infection is associated with lymphoproliferative disorder (LPD) and is predominantly seen with T cell depleting regimens, HLA mismatched donors, EBV mismatch, splenectomy, and umbilical cord blood transplants [71].

Different clinical patterns of lymphoproliferative disorders have been described: (i) Acute illness due to infectious mononucleosis is seen in the first two months after induction, typically characterized by polyclonal B cell proliferation; (ii) Neoplastic pattern with early malignant transformation with cytogenetic abnormalities and immunoglobulin gene rearrangements; (iii) Post-transplant lymphoproliferative disorder is characterized by fever, lymphadenopathy, and multi-organ involvement, including nodular pulmonary infiltrates [72].

Other Herpes Viruses

Acyclovir or valacyclovir prophylaxis has significantly reduced the incidence of HSV pneumonitis [67]. Recent studies indicate that the presence of HHV-6 isolated from lower respiratory tract samples is linked to higher mortality rates and acute non-infectious lung injury. Common symptoms include fever, cough, and shortness of breath, often accompanied by diffuse infiltrates [73].

Bacterial Pneumonia

The reported incidence of bacterial pneumonia in solid organ transplants is 7-11%, increasing to 16% during the first month after lung transplantation. Late bacterial infections occur at an incidence of 15%, even years post-HSCT [33]. Respiratory infections from encapsulated bacteria like *Pneumococcus*, *Haemophilus influenzae*, and *Neisseria meningitidis*, present predominantly as sino-pulmonary infections, particularly during the late post-engraftment period [33,61].

Chronic GVHD treatment can lead to deficiencies in IgG2 and IgG4, increasing susceptibility to infections from encapsulated organisms, which are mostly observed in the late post-engraftment phase. Pneumococcal infections can occur despite vaccination and Amoxicillin prophylaxis, with a mean onset of around 473 days post-HSCT. Additionally, Gram-negative pathogens, such as Pseudomonas aeruginosa, are frequently isolated in both early and late periods after lung transplantation [24,61].

Risk factors for bacterial pneumonia include prolonged mechanical ventilation, a diminished cough reflex, and impaired mucociliary clearance. Organisms found in donor bronchial washings do not reliably predict pneumonia in lung transplant recipients, even among CF patients with Pseudomonas aeruginosa. However, the isolation of *Burkholderia cepacia* (especially the genomovar III variant) from donor lungs has been linked to severe infectious complications in recipients, leading to one-year survival rates of 50-67% compared to 83-96% without this organism [61,74].

Nocardia

This infection is mainly observed in the late post-engraftment stage, presenting with symptoms suggestive of pneumonia, cerebral infarcts, and skin lesions [50].

PJP Pneumonia

PJP pneumonia carries a high mortality rate among transplant recipients. Prophylaxis with trimethoprim-sulfamethoxazole (TMP-SMX) has reduced the incidence of PJP to about 0.63% in allogeneic and 0.28% in autologous HSCT cases [37,75].

Risk factors for PJP in HSCT include prolonged steroid use, lymphopenia, rituximab therapy, mismatched grafts, chronic GVHD, and non-compliance with prophylaxis. Inadequate prophylactic measures, such as aerosolized pentamidine or dapsone administered thrice weekly, heighten the risk of *Pneumocystis* infection [76]. For patients receiving prolonged high-dose steroids or those with a CD4 count below 200 cells/uL, *Pneumocystis* prophylaxis should always be considered [37].

In solid organ transplantation, prophylaxis is typically administered for one year before discontinuation. However, lung transplant recipients often receive PJP prophylaxis indefinitely. Risk factors for PJP in single organ transplant patients include older age, lymphopenia, and previous CMV infection [77].

Patients with PJP commonly manifest high fever, dry cough, hypoxia, and shortness of breath, accompanied by interstitial infiltrates.

Fungal Infections

Fungal infections encompass endemic mycoses, yeast infections like *Candida*, and invasive mold infections.

The incidence of invasive fungal infections reaches 5-30% in allogeneic HSCT and up to 5% in autologous HSCT [78]. In the early post-engraftment phase, 40-50% of nodular pneumonia cases and around 90% of pulmonary fungal infections result from invasive aspergillosis, while 5-10% stem from mucormycosis [79]. Among solid organ transplants, lung transplant patients exhibit the highest incidence of invasive pulmonary aspergillosis, with a one-year cumulative incidence of 2.4%, versus 0.1% in kidney transplants. Despite prophylactic and anti-fungal treatments, mortality rates for invasive pulmonary aspergillosis can reach 75%, with 67% in heart and kidney transplant recipients and 20% in lung transplant recipients [80]. Infections due to mucorales and fusarium also pose a significant mortality risk in transplant patients [20].

Invasive mold infections primarily include *Aspergillus fumigatus* (the most common), Mucorales such as *Rhizopus*, *Mucor*, and *Rhizomucor*, as well as *Fusarium*, *Lomentospora*, and *Scedosporium*.

*Aspergillus*, a widespread saprophytic fungus, thrives on decaying organic matter. Its hyphal forms and conidia are present in air conditioning units and flood-damaged homes. Environmental activities like gardening and construction also elevate the risk of invasive aspergillosis. Various *Aspergillus* species can lead to invasive infections, including *A. fumigatus*, *A. flavus*, *A. terreus*, *A. niger*, and *A. nidulans* [81].

*A. fumigatus* is the primary cause of invasive pulmonary aspergillosis, while *A. flavus* and *A. niger* are linked to sinus infections. HEPA (high-efficiency particulate air) filters appear to reduce the concentration of conidial spores, potentially offering protection [81]. Some fungal infections, such as mucorales, can manifest with sinonasal and pulmonary symptoms [82].

Several risk factors are associated with invasive aspergillosis, including GVHD (both acute and chronic), GVHD therapies like Ibrutinib, CMV disease, prolonged neutropenia, allogeneic grafts, and myeloablative conditioning regimens. Recently, conditions such as COPD (chronic obstructive pulmonary disease), HIV (human immunodeficiency virus), and post-viral infections-including influenza and SARS-CoV-2-have also been identified as risk factors [83]. Neutropenia and macrophage dysfunction induced by corticosteroids significantly contribute to invasive *Aspergillus* infections. In contrast, alterations in T cell defenses lead to allergic and chronic infections. Notably, a history of CMV infection can modify cellular immune responses, increasing the likelihood of invasive aspergillosis. Previous use of voriconazole, iron overload, and elevated environmental temperatures raise the risk of mucormycosis [82].

Invasive fungal infections typically present with high fever, shortness of breath, dry cough, hemoptysis, and pleuritic chest pain. However, in immunosuppressed individuals, the only signs might be an asymptomatic nodule or a solitary fever. Clinical presentations can vary significantly and depend largely on the host's immune response, with severe invasive disease observed in individuals with more pronounced immune deficiencies.

For a post-lung transplant patient experiencing cough, wheezing, stridor, or dyspnea with normal radiographs, clinicians should consider *Aspergillus tracheobronchitis*, which may present in one of three patterns: (i) Obstructive due to thick mucus plugs filled with *Aspergillus hyphae*; (ii) Ulcerative affecting either the mucosa or cartilage, or (iii) Pseudomembranous types caused by necro-inflammatory debris.

In lung transplant recipients, tracheobronchitis from invasive aspergillosis may lead to dehiscence of bronchial anastomosis. Notably, frank hemoptysis is apparently a late symptom of tracheobronchitis [84].

Other signs can include CNS or GI diseases and skin nodules.

Fever and pulmonary infiltrates with hemoptysis in the setting of neutropenia should strongly raise suspicion for invasive pulmonary aspergillosis (IPA), especially if there’s no improvement with broad-spectrum antibiotics. Mucorales should be suspected if sinus symptoms are present. Certain fungal infections like *Fusarium* may also present with skin lesions, warranting blood cultures in such scenarios [85].

Aspergillosis can present in one of the four clinical syndromes [81]. (i) Allergic aspergillosis is primarily driven by a vigorous CD4 Th-2 response and as the name suggests, this is primarily an allergic manifestation and carries no risk of invasive disease. This allergic syndrome manifests as asthma, allergic sinusitis, and allergic bronchopulmonary aspergillosis (ABPA). (ii) Aspergillomas or mycelial fungal balls develop in damaged lung tissue areas like bronchiectasis or existing cavities. These fungal balls alone do not pose a risk of invasive disease [86]. (iii) Sub-acute semi-invasive aspergillosis occurs in individuals with mild to moderate immune impairment, such as those with diabetes, on steroids, or with pulmonary disease. Typically, this presents as a localized disease with cavitary or fibrosing manifestations. (iv) The most serious form, invasive aspergillosis, is seen in markedly immunocompromised patients, such as those with neutropenia or transplant recipients. Commonly affected organs include the sinuses and lungs, with potential dissemination to remote organs such as the skin, brain, spleen, and kidneys. Rhino-sino-cerebral aspergillosis can exhibit sino-pulmonary and cerebral symptoms [20,87].

Mycobacterial Infections

Mycobacterial infections occur in 1-3% of allogeneic stem cell transplant (HSCT) patients and 0.2% of autologous HSCT cases, usually due to either reactivation or new infections. Reactivation of tuberculosis can happen years post-transplant [88,89]. These infections may show a range of symptoms and a wide variety of radiographic infiltrates, emphasizing the importance of risk assessment and exposure history in their evaluation.

Strongyloides Infections

Disseminated Strongyloides can manifest as ARDS (acute respiratory distress syndrome) and may be accompanied by gram-negative sepsis and pneumonia. Thus isolation of Gram-negative bacteria in the blood may not completely explain the clinical syndrome and further testing should always be considered. A history of exposure to contaminated soil can raise suspicion for this rapidly progressing, potentially fatal hyper-infection, although reactivation after many years has also been documented post-transplantation [48].

Diagnosis and evaluation

Given the overlap in clinical signs and imaging characteristics, as well as the common occurrence of co-infections, it is essential to pursue a specific diagnosis whenever possible. The evaluation of suspected pneumonia in transplant recipients depends on symptom severity, the risk of opportunistic infections, the level of immunosuppression, and exposure history. In cases with less severe presentation and low risk for opportunistic infections, a limited evaluation may suffice for empiric therapy. However, in severe instances, invasive diagnostic methods, such as lung biopsies along with broad-spectrum antimicrobial treatment, are typically necessary. A low threshold for conducting diagnostic tests for pneumonia in transplant patients is recommended. Bronchoscopy with broncho-alveolar lavage is the preferred technique, especially if done within 24 hours of symptom onset before initiating empiric antimicrobial therapy. Given the risk of clinical deterioration in these patients, early proactive diagnostic testing is vital instead of relying solely on an empiric or watchful approach [61].

Lab Testing

Routine basic lab tests, including a complete blood count with differential, comprehensive metabolic panel (evaluating renal and liver function), and chest radiography, are necessary for all transplant patients suspected of pneumonia. These tests not only aid in diagnosis but also assess the toxicity risk associated with antimicrobial therapy [90].

In post-transplant patients, serological antibody tests are often inadequate due to immunosuppressive treatments that can delay or abate seroconversion. Nonetheless, serology may provide insights into past exposures and risks during pre-transplant assessments. Thus culture or histopathology-based diagnostic methods are preferable. Antigen-based or nucleic acid testing should also be utilized when available to corroborate specific diagnoses [91].

While urinary antigen tests for *Legionella* have low sensitivity and miss some species, they are advised due to their non-invasive approach and cost-effectiveness [92]. A positive outcome can lead to quicker diagnosis and minimize the need for invasive procedures. Additionally, multiplex molecular respiratory viral panels or nasopharyngeal swabs for influenza PCR and other seasonal respiratory pathogens should be used as appropriate [62]. If possible, sputum gram staining and cultures should be obtained, and blood cultures should be performed for febrile patients or those requiring hospitalization [2].

Further diagnostic assessments should be customized according to symptom severity, individual opportunistic infection risk, and local epidemiological trends. This could involve TB-IGRA (tuberculosis - interferon-gamma release assay) for tuberculosis, serological or antigen tests for endemic fungi, fungal biomarker assays like galactomannan or beta-D-glucan, and CMV Q NAT (cytomegalovirus virus quantitative nucleic acid testing). Elevated galactomannan levels, paired with specific radiographic findings, may signal an invasive fungal infection. CMV testing must be tailored to the donor-recipient CMV status, immunosuppression level, time since transplantation, and CMV prophylaxis status, given that solid organ transplant recipients are most vulnerable to invasive CMV infections when donors are seropositive and recipients are seronegative [61].

Although procalcitonin levels can aid in distinguishing bacterial infections, the evidence in organ transplant patients is limited. An elevated procalcitonin level beyond the first week post transplantation may indicate a bacterial infection, but factors such as surgery and anti-thymocyte globulin administration can affect the results. Caution is warranted in interpreting procalcitonin levels within the first week, and lack of elevation should not rule out bacterial infections until more evidence is accumulated [93].

It is also vital to recognize that pneumonia severity scores like CURB-65 and Pneumonia Severity Index, which are validated for community-acquired pneumonia, yield poor results in immunosuppressed patients, particularly those with cancer and potentially can be extrapolated to transplant recipients [94].

Bronchoscopy

Bronchoalveolar lavage (BAL) yields vary between 39% and 77% and imaging studies can aid in targetting specific segments for BAL. Specimens obtained from BAL should be cultured for bacteria, fungi, mycobacteria, and viruses, and subjected to AFB (acid-fast bacilli), silver, and GMS (Grocott's methenamine silver) stains for evaluating bacterial, fungal, and PJP infections. It is advisable to test for antigens such as galactomannan or endemic fungi, and if available PCR for respiratory viruses, CMV, *Mycoplasma*, *Legionella*, *Ureaplasma*, PJP, *Nocardia*, and invasive molds. PJP testing should prioritize high-risk patients who are not on prophylaxis. Conducting multiplex PCR on BAL specimens is recommended to identify lower respiratory tract infections, even when a nasopharyngeal swab has confirmed the diagnosis, as co-infections are common in transplant recipients [95].

While biomarkers for fungal infections like galactomannan and 1,3 beta-D glucan have been utilized in hematopoietic stem cell recipients, their effectiveness in solid organ transplant patients is comparatively limited [96].

Lung Biopsy

If a patient's condition worsens despite 24-48 hours of empiric therapy, with non-diagnostic initial evaluations, or if the initial presentation is severe, it is crucial to obtain additional samples via bronchoscopy, alveolar lavage, and if necessary, lung biopsy. The decision to perform a lung biopsy hinges on confidence in the diagnosis, the outcomes of other tests, and the procedure's risk versus benefit.

Clinical scenarios that necessitate a lung biopsy include (i) a patient showing diffuse or focal lung infiltrates who does not improve after empiric antibiotic therapy and lacks a confirmed diagnosis from non-invasive tests, including broncho-alveolar lavage; and (ii) patients with focal nodular infiltrates who are at high risk for fungal infections or malignancies, such as post-transplant lymphoproliferative disorder.

Conversely, if a patient resides in areas with high endemic fungus exposure, it may be reasonable to wait until serological and antigen testing is completed. A negative serological and antigen result does not rule out fungal infections, and a lung biopsy might ultimately be necessary [97].

Collecting tissue specimens is particularly beneficial for diagnosing fungal and viral infections and malignancies. CT scans of the chest can aid in pinpointing the biopsy site. Lung biopsies can be performed via transbronchial approach, IR (interventional radiology) guidance, VATS (video-assisted thoracic surgery), or open lung biopsy.

One study indicated a 53% diagnostic yield for CT-guided lung biopsies, with a 13% risk of complications. Open lung biopsies have an approximate 85% diagnostic yield but come with a complication rate of 28%, including mortality. Nonetheless, lung biopsies have led to a change in therapeutic management in 33% to 53% of cases [98]. If performed, tissue specimens should be sent for culture (bacterial, fungal, viral, AFB), stains for fungal and PJP, and molecular studies for infectious agents. Selected facilities offer broad-spectrum PCR detection of infectious agents, including 16sRNA, pan-fungal, and mycobacterial genus PCR or next-generation sequencing. However, further studies are needed to define the utility and effectiveness of these tests [99].

Radiographic Imaging

CT chest should be strongly considered if the presentation is moderate to severe or if the degree of immunosuppression is high. Conventional radiographs lack sensitivity and specificity for detecting pneumonia in immunosuppressed patients. The gold standard for pneumonia diagnosis in transplant recipients remains microbiology, with histology often being necessary [100]. Thus, significant confidence should not be placed solely on radiological signs due to substantial overlap, though certain features may raise suspicion for specific etiological agents. Sometimes, the same organism may have different radiographic manifestations depending on the level of immunosuppression. For instance, invasive pulmonary aspergillosis in HSCT typically presents with a nodular-consolidative pattern, unlike peribronchial opacities seen in solid organ transplant recipients [80]. Bacterial or mold infections tend to cause nodular patterns, whereas PJP, viral infections (CMV, other respiratory viruses), or non-infectious pneumonia usually manifest as diffuse infiltrates. Sometimes, radiographic patterns and timeline post-transplant can help narrow the differential diagnosis [101]. For example, during the pre-engraftment period, nodular infiltrates are usually infectious, while diffuse infiltrates may stem from non-infectious causes.

Certain classic radiographic signs have been identified, such as the “halo sign” during the pre-engraftment stage, which is suggestive of invasive pulmonary aspergillosis, while the “reverse halo sign” or “bird nest sign” suggests mucormycosis [82]. Although these signs are described as “sine qua non”, there is significant overlap, and corroborative microbiological evidence should be sought based on the circumstances. Mycobacterial infections can show various infiltrates and should be suspected based on risk and exposure assessment [55].

The radiographic patterns of infectious pneumonia and their differential diagnoses are outlined below [101]. A combination of findings increases the likelihood of identifying the specific agent responsible for pneumonia [78]. (i) Diffuse or patchy ground-glass opacities: (a) community-acquired respiratory viruses [62], (b) herpes viruses [67], (c) *Pneumocystis jiroveci* pneumonia [77], (d) disseminated *Strongyloides *[48]; (ii) Diffuse or patchy consolidation: (a) herpes viruses [67], (b) invasive pulmonary aspergillosis in HSCT [79], (c) bacterial pneumonia [101], (d) disseminated *Strongyloides* [48]; (iii) Tree-in-bud opacities: (a) community-acquired respiratory viruses [94], invasive fungal infection in solid organ transplant (SOT) [20]; (iv) Solitary or multiple nodules (focal or diffuse nodular): (a) herpes viruses [67], (b) invasive pulmonary aspergillosis in HSCT [78], (c) bacterial pneumonia [101], (d) *Nocardia* [50]; and (v) Predominantly peri-bronchial: (a) invasive pulmonary aspergillosis in SOT [80].

Final Diagnosis

Community-acquired respiratory viruses [94]: Radiographic images might reveal diffuse or patchy ground-glass opacities, consolidation, or tree-in-bud patterns. If a community-acquired respiratory virus infection is suspected, multiplex PCR panels are typically conducted on nasopharyngeal swabs. Regardless of the test outcomes, it is recommended to perform a bronchoscopic diagnosis using multiplex PCR on BAL fluid. This approach is advised due to the potential for co-infections with various pathogens, especially in transplant recipients, and because the organisms detected in upper respiratory samples do not always match the findings in the lower respiratory tract [61].

Herpes viruses [67]: Radiographic patterns may encompass diffuse or patchy ground-glass opacities, consolidative infiltrates, and solitary, focal, or diffuse nodules. The presence of nodular infiltrates during the late post-engraftment period strongly suggests an infection due to EBV [72]. It is important to note that herpes viruses are excluded from the multiplex PCR testing designed for community-acquired respiratory viruses [102].

Cytomegalovirus (CMV) [47]: CMV infection, particularly presenting as viremia or antigenemia, is identified through PCR or p65 antigen assays. Viral shedding is typically confirmed by isolating CMV in BAL specimens. However, the mere identification of CMV in BAL (via culture or PCR) cannot differentiate between active infection and asymptomatic shedding. Cytopathological alterations revealed by CMV-specific stains during lung biopsy are needed to diagnose CMV pneumonia. In certain instances, patients are on mechanical ventilation with high-pressure settings which may preclude biopsy for safety reasons. In those circumstances, BAL cytology demonstrating cytopathological changes can provide corroborative evidence for CMV pneumonia. Nonetheless, both lung biopsy and BAL cytology may lack sufficient sensitivity and fail to identify cases of the disease [56]. Consequently, a multifaceted diagnostic approach becomes essential.

*Pneumocystis jirovecii* pneumonia (PJP): *Pneumocystis* typically presents as diffuse infiltrates, with elevated serum beta-D-glucan levels, particularly among transplant recipients. Diagnosis is routinely confirmed through direct immunofluorescent staining or PCR of preferable lower respiratory samples [77].

Invasive mold infections: As previously noted, invasive pulmonary aspergillosis manifests with different radiographic patterns in HSCT and SOT patients. In HSCT cases, a nodulo-consolidative pattern manifests, while peri-bronchial or tree-in-bud (TIB) opacities are observable in SOT patients. Radiographic findings may vary in accordance with levels of immunosuppression and can fluctuate during treatment. A common presentation of invasive pulmonary aspergillosis during the pre-engraftment phase is the “halo sign,” characterized by a nodule enveloped by ground-glass opacities. Occasionally, during treatment, increasing neutrophil counts may lead to nodules enlarging in size and number or even undergoing cavitation [79]. A bird nest or reverse halo sign, defined by ground-glass opacity surrounded by consolidation, is indicative of mucormycosis [103].

Serum galactomannan exhibits reasonable sensitivity for diagnosing invasive aspergillosis in HSCT patients when compared to those with solid organ transplants. Galactomannan levels can also be evaluated through bronchoscopy-alveolar fluid. Serum beta-D-glucan serves as a marker for invasive fungal infections, albeit its sensitivity is limited for certain dimorphic fungi and mucormycosis. PCR from blood and BAL is increasingly utilized for the diagnosis of invasive fungal infections [78]. Microbial evidence and histological findings may be necessary to confirm invasive aspergillosis. Additionally, serum quantitative PCR is now available for diagnosing mucormycosis. Once mucormycosis is identified, radiographic assessment for metastatic spread to the brain and spleen is important. Cases of co-infection with mucormycosis and aspergillosis have also been documented [82].

Treatment and management

The selection of empiric antimicrobial therapy is contingent upon the suspected pathogen responsible for pneumonia [61]. Generally, an empiric antibiotic approach is favored over a conservative strategy. The selection of antibiotic therapy must consider previous colonization patterns and antimicrobial resistance profiles. Drug interactions further play a critical role in the selection of antimicrobials. During the influenza season, antiviral therapy should be administered in conjunction with antibiotics unless rapid PCR testing is available [63]. Empiric anti-fungal therapy is warranted in scenarios where there is a strong clinical and radiological suspicion of fungal pneumonia. Adjustments to empirical therapy may be necessary based on clinical progression and microbiological results. Most empirical treatments do not target infections caused by *Nocardia*, *Pneumocystis jirovecii*, mycobacteria, or fungi. Aggressive diagnostic testing, such as bronchoalveolar lavage or tissue sampling, must be conducted if risk factors for these pathogens are recognized [100].

Respiratory Viruses

Currently, specific treatments for community-acquired respiratory viruses remain unavailable. If influenza is suspected, neuraminidase inhibitors, such as oseltamivir, should be promptly administered, ideally within 48 hours after the onset of symptoms [63]. Should patients continue to deteriorate despite antiviral therapy, it becomes crucial to evaluate for oseltamivir resistance. Oral or inhaled ribavirin has been suggested for respiratory syncytial virus infections; however, the evidence supporting its efficacy is limited [104]. Paxlovid has received approval for high-risk patients diagnosed with COVID-19. Furthermore, dexamethasone has been shown to reduce mortality in COVID-19 patients exhibiting hypoxia [94,105].

CMV

The treatment with ganciclovir or valganciclovir has significantly reduced mortality rates. Typically, CMV disease is treated with intravenous ganciclovir administered over two to three weeks at a dosage of 5 mg/kg twice daily, with dosage adjustments made based on estimated glomerular filtration rate (eGFR). Due to its poor bioavailability, oral ganciclovir is not recommended [106]. Although oral valganciclovir possesses good bioavailability, many physicians still prefer intravenous ganciclovir owing to the lack of robust evidence supporting oral valganciclovir. In very severe cases, the selective use of CMV hyperimmune globulin may be warranted on a case-by-case basis [47].

The relapse rate for CMV disease is notably high, ranging between 20-60%. Reports of ganciclovir-resistant CMV are increasing, with an incidence approaching 5%, which corresponds to diminished survival rates [107]. Risk factors for resistant CMV include the use of anti-lymphocyte antibodies, daclizumab, and the frequency of CMV infections. Foscarnet is the preferred treatment for ganciclovir-resistant CMV; however, its use is often associated with significant nephrotoxicity [108].

Considering the high mortality and morbidity associated with CMV infections post-transplant, proactive prevention is typically the most effective strategy to mitigate complications. Ideally, seronegative donors and blood transfusions should be considered for seronegative organ recipients, particularly in the context of solid organ transplantation. Nevertheless, as the majority of the donor pool is seropositive, alternative strategies, such as pharmacological prophylaxis with ganciclovir, are implemented to delay the onset and reduce the incidence and severity of infections. Two prophylactic approaches are commonly practiced: universal prophylaxis entails administering ganciclovir for three months post-transplant for any seropositive donor or recipient, while preemptive prophylaxis utilizes ganciclovir based on serial monitoring of asymptomatic viremia through p65 testing or PCR assays [47,109].

Bacterial Pneumonia

If the patient is deemed low risk for opportunistic or hospital-acquired infections in the outpatient setting, beta-lactams, macrolides, or fluoroquinolones may be considered to cover community-acquired respiratory pathogens. It is advisable to include empirical coverage for intracellular pathogens such as mycoplasma, chlamydia, and legionella [61]. Caution should be exercised regarding potential drug interactions and community resistance patterns. In circumstances involving significant interactions with immunosuppressive medications, or in cases of increasing resistance of mycoplasma to macrolides, fluoroquinolones may be pursued based on a careful consideration of risk versus benefit [110]. Empirical coverage for MRSA and *Pseudomonas* is dictated by local prevalence, resistance patterns, and the patient’s colonization history.

Upon hospitalization, the selection of antibiotic therapy is influenced by whether the pneumonia is hospital-acquired or ventilator-associated, with an assessment of risk factors for MRSA or *Pseudomonas*. Notable risk factors for hospital-acquired infections include recent hospitalizations, chronic lung diseases such as bronchiectasis, or the presence of prosthetic devices, which necessitate coverage for gram-negative pathogens [54].

PJP Pneumonia

The first-line therapy for PJP is intravenous TMP-SMX. For patients with sulfa allergies, alternatives such as clindamycin/primaquine are available. In severe cases, adjunctive steroids are recommended. Prophylactic options include TMP-SMX, atovaquone, dapsone, or pentamidine [75].

Invasive Fungal Infections

High-risk patients, such as those who have undergone HSCT with severe GVHD, are typically placed on prophylaxis with posaconazole or voriconazole. Low-risk patients generally receive fluconazole prophylaxis [78]. Lung transplant recipients may receive inhaled liposomal amphotericin as preemptive prophylaxis during the early post-transplant phase [111]. Despite these preventive measures, invasive fungal infections remain a risk; therefore, microbiological diagnosis should be pursued upon suspicion.

*A. terreus* may exhibit resistance to amphotericin, while *A. nidulans* and *A. calidoustus* may resist multiple anti-fungal therapies, including voriconazole and amphotericin B [112].

Voriconazole or isavuconazole is commonly utilized as first-line treatment for invasive aspergillosis; posaconazole may serve as salvage therapy. Liposomal or lipid complex amphotericin B can be administered to patients who are intolerant to triazole or who experience breakthrough infections while on triazole prophylaxis. The increasing prevalence of azole-resistant *Aspergillus* necessitates consideration if clinical improvement remains elusive despite treatment [80].

Liposomal amphotericin B is the preferred treatment for mucormycosis; however, isavuconazole is gaining popularity. Consultation with cardiothoracic surgery for potential surgical intervention is crucial for all patients diagnosed with mucormycosis. Following surgery and initial amphotericin treatment, patients may transition to step-down therapy utilizing posaconazole or isavuconazole [113]. Consultation with infectious disease specialists is invaluable for the management of these cases [25].

## Conclusions

Patients who have undergone transplants face an increased risk for a variety of typical and atypical microbiological infections. These infections can lead to significant morbidity and mortality. The implementation of prophylactic measures is essential, as prevention is certainly more effective than treatment. Immunocompromised transplant patients may not exhibit typical signs or symptoms of infection, necessitating a heightened index of suspicion, with early initiation of broad-spectrum anti-infective measures and aggressive diagnostic testing, including lavage, biopsy, serological, and molecular methods. Multi-disciplinary collaboration and co-management are critical. Despite advancements in medical science, infections continue to significantly affect the outcomes for many transplant patients.
